# Estimation of the public radiation dose from ¹⁷⁷Lu in wastewater discharged by nuclear medicine centers in Tehran, Iran

**DOI:** 10.22038/aojnmb.2026.89390.1648

**Published:** 2026

**Authors:** Mohammad Reza Deevband, Mohammad Reza Asgari, Mahasti Amoui, Mohammadali Ghodsirad

**Affiliations:** 1 Biomedical Engineering and Medical Physics Department, School of Medicine, Shahid Beheshti University of Medical Sciences Tehran, Iran; 2 Nuclear Medicine Department, Shohada Tajrish Hospital, School of Medicine, Shahid Beheshti University of Medical Sciences, Tehran, Iran

**Keywords:** Public dose, Lutetium therapy, Prostate cancer, Neuroendocrine tumors Radionuclide therapy

## Abstract

**Objective(s):**

Currently, the treatment of prostate and neuroendocrine tumors with radiopharmaceuticals derived from lutetium-177 is the focus of many radionuclide therapy centers. One of the reasons for turning to lutetium radiopharmaceuticals is their favorable dosimetry and stability. Directly excreting the urine of patients treated with these radiopharmaceuticals into the hospital's waste water and consequently into the city's waste water and finally into agricultural fields causes indirect radiation exposure. This study aims to calculate the radiation exposure of people in Tehran due to the presence of this radioactive material in the wastewater of urban areas.

**Methods:**

First, the activity concentration in wastewater was estimated, and then, the dose received by people was estimated via a mathematical model.

**Results:**

The amount of public dose of lutetium in wastewater from nuclear medicine centers in Tehran is 8712.9 nSv/year.

**Conclusion:**

The direct release of patients' urine into the wastewater of nuclear medicine centers in Tehran does not have a significant effect on the radiation exposure of people, and septic tanks are not necessary for these radiopharmaceuticals.

## Introduction

 One application of nuclear medicine is prostate cancer treatment. Prostate cancer is one of the most common malignancies in men and is the main cause of cancer death worldwide (1). Many nuclear medicine centers around the world have confirmed that lutetium (Lu) has favorable dosimetry and a convincing therapeutic response to prostate cancer (2). 

Table 1 shows the physical parameters of the lutetium.

**Table 1:** Physical features of lutetium

**Isotope**	**Half life**	**Gamma energy** **(Kev)**	**Maximum beta energy** **(Kev)**	**The mean of beta energy** **(Kev)**	**Maximum beta ranges in tissue** **(mm)**	**The mean of beta ranges in tissue** **(mm)**
Lutetium	6.6 days	113 and 208	498.3	134.2	2	0.5

 Prostate cancer treatment with lutetium is a safe method that is approved by physicians (3). One of the applications of lutetium is neuroendocrine tumors treatment. ^177^Lu-PSMA is used to treat prostate cancer, and ^177^Lu-DOTATATE is used to treat neuroendocrine tumors (4). The results obtained from the treatment of patients suffering from neuroendocrine tumors demonstrate that treatment with this radiopharmaceutical has been very effective and has improved the quality of life of patients (5). Patients who are treated with lutetium are quarantined in the nuclear medicine department for 4 hours after the end of the treatment process (6). This is due to the radiation protection of the treatment staff and people who are around the patient (7).

**Figure 1:**
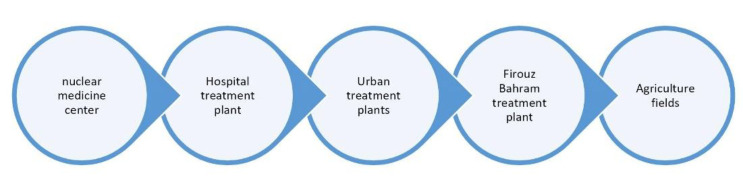
The route of waste water from the nuclear medicine department to agricultural fields

 Some of the radioactive materials injected into patients are excreted through urine during quarantine. As shown in Figure 1, this urine first travels through the wastewater system to the hospital treatment plant and then enters the city sewer. Then, it enters agricultural fields and agricultural products through surface water. The wastewater of the examined medical centers is poured into various treatment plants in the city, but all these treatment plants are finally discharged to the Firouz Bahram treatment plant, which provides 190 million cubic meters of water for the agricultural sector.

 Eating these agricultural products causes people to be exposed to radiation. In the present study, the amount of public radiation exposure due to the activity released by patients treated with lutetium into wastewater was calculated. Obtaining the level of radiation exposure of people from this type of radiopharmaceutical will improve the health level of society in terms of radiation protection.

## Methods

The target population in this study is the residents of Tehran, who have been indirectly exposed to the radiation of lutetium radiopharmaceuticals. There are 16 nuclear medicine centers in Tehran that use radiopharmaceuticals derived from lutetium to treat patients with prostate and neuroendocrine tumors.

 The average amount of radiopharmaceuticals given to each center is 1000 mCi per month. Considering that an average of 200 mCi (4007MBq) of radiopharmaceutical is needed to treat each patient, the number of patients treated each month in Tehran is 80 (8).

 The wastewater of these treatment centers is first poured into the center's own treatment plant, then flows into the intermediate treatment plant located in the city, and finally, all these treatment plants are discharged to the Firouz Bahram treatment plant. This treatment plant is located southwest of Tehran, and its capacity is 6.5 m^3^ per second. Ethical considerations and approval from the relevant institution were considered for access to relevant data.

 Using the following mathematical model, the dose received by people was estimated(9).

E_d_= A_c_ × C_a_ × D_coff (1)_

 E_d_ is the annual effective dose for people in nSv, A_c_ is the activity concentration in water in Bq/L, C_a_ is the per capita water consumption in liters (L), and D_coff_ is the dose coefficient provided for lutetium by the ICRP, which is equal to 0.643 nSv (10).

 Considering that patients excrete half of the injected radioactive substance through urine during quarantine and with the knowledge of the water volume of the destination treatment plant, the amount of activity in the wastewater was calculated (6). The per capita consumption of water per person during the day is equal to 8 glasses or 2 liters (11).

 The dose limit for people receiving a specific radiation is 10 µSv per year (12). In other words, the amount of radiation exposure of people from lutetium in wastewater, which is considered special radiation, should not be more than 10 µSv.

## Results

 The number of patients who are treated with lutetium radiopharmaceuticals in one month in Tehran is 80. On the other hand, the average number of injected radiopharmaceuticals per patient is 200 mCi (4007 MBq). With knowledge that half of the injected radioactive substance is excreted through urine during the quarantine of the patient, we conclude that 8000 mCi (296000 MBq) is released from all these patients and enters the wastewater cycle.

 The effluent is diluted in the treatment plant of the nuclear medicine center and subsequently diluted to the city treatment plant. However, owing to the lack of accurate data on the volume of these treatment plants and to obtain the maximum dose received by the people, we abandoned the dilution of the wastewater in these treatment plants. The volume of water entering Firouz Bahram's water treatment plant in one month is equal to 15724800 m^3 ^(15724800000 L).

 With the dilution of radioactivity in Firouz Bahram’s water treatment plant, the concentration of activity becomes 18.82 Bq/L. The per capita water consumption of a person in a month is equal to 60 liters. According to the mentioned mathematical model, the dose received by people in a month is equal to 762.07 nSv, and that in a year is equal to 8712.9 nSv.

 Assuming that a person supplies all the water consumed per capita from Firouz Bahram's water treatment plant, the dose received by that person from the activity of lutetium in the water will be equal to 8712.9 nSv/year.

## Discussion

 The maximum radiation exposure of people in Tehran from the activity of lutetium in wastewater is 8712.9 nSv/year.

 The use of lutetium radiopharmaceuticals is highly beneficial for the treatment of prostate and neuroendocrine tumors (13). However, the point to consider when these radiopharma-ceuticals are used is the direct release of treated patients' urine into wastewater. Radioactive substances in waste water indirectly cause radiation to people. For this reason, the lack of knowledge about this amount of radiation has caused few patients to be treated in nuclear medicine centers. The results of this study confirm that the amount of radiation exposure of people from the lutetium in the effluent from the nuclear medicine centers of Tehran does not cause the radiation of the people of this city to exceed the permissible limit. Therefore, there is no need for a septic tank to store this waste of radioactive material.

 In this study, a mathematical model was used to estimate the dose received by people. Using this method, the maximum dose reached to people was calculated. It has been assumed that people will consume all their water per capita from the Firouz Bahram treatment plant; however, the amount of wastewater diluted in the treatment plants of nuclear medicine centers and urban treatment plants has been ignored. Additionally, after the levelling of water and agricultural lands and being absorbed by plants, the activity of the radioactive substance decreases over time.

 Martin GE and colleagues in 1997 (14) investigated the amount of radiation exposure of people from the iodine in the wastewater released by a medical center via a mathematical model and reported that the amount of radiation exposure of the people from the iodine in the wastewater is negligible and can be ignored. One of the reasons for the low dose can be attributed to the high dilution of the activity in the wastewater. The volume of water is very large compared with the amount of activity. It seems that only if a large amount of activity enters wastewater in a short time can it greatly increase the public dose.

 In 2010, Fernando Jimenez and colleagues in Spain (15) measured the activity of iodine-131 in wastewater via the liquid scintillation technique via direct sampling at several points and reported that the dose received by the public was lower than the annual limit.

 In the present study, due to the limitations associated with laboratory use, a mathematical model was used to estimate the dose to the public. Therefore, the use of the liquid scintillation method to determine the activity in wastewater is recommended in future studies to increase the accuracy of the obtained results.

 In 2013, MC Gowan and colleagues(16) investigated and measured iodine-131 in wastewater and reported that the dose estimated via the direct sampling method was much lower than that via the mathematical model method.

 Therefore, one of the reasons that it is claimed in the present study that the maximum dose reached to the people was calculated is the use of a mathematical model instead of direct sampling.

 To date, 80 patients in Tehran have been treated with lutetium radiopharmaceuticals; with the knowledge that it is safe to directly release the treated patient’s urine into wastewater, the number of these patients can be increased. According to the obtained results, the number of treated patients per month can be increased to 90 patients without exceeding the permissible dose limit.

 This will reduce the waiting time of patients, and by using lutetium radiopharmaceuticals in other cities, patients will not have to travel to another place for treatment. In this study, due to the lack of access to the gamma spectrometry laboratory, direct sampling of wastewater was not performed, so direct wastewater sampling and measurement of the radioactive material content via gamma spectrometry were suggested to increase the accuracy of the results. This study can also be performed for other cities. Therefore, further studies using direct measurement methods (gamma spectrometry) are recommended to validate these findings.

## Conclusion

 The amount of radiation exposure of people from lutetium in the effluent from nuclear medicine centers in Tehran is equal to 8712.9 nSv/year, which is less than the allowed limit (10000 nSv/year). Therefore, the direct release of urine from patients treated with lutetium radiopharmaceuticals into wastewater is unimpeded and does not have a significant effect on radiation exposure.
